# Efficacy of Fatigue Countermeasures on Cognitive Performance and Operational Safety in Military Pilots: A Systematic Review

**DOI:** 10.7759/cureus.95823

**Published:** 2025-10-31

**Authors:** Abdullah Attiah Alzahrani, Meshal F Alessa, Fahad Musaad Alharbi, Talal Mohammed Alotaibi

**Affiliations:** 1 Family Medicine, Northwest Area Command, Royal Saudi Land Forces, Ministry of Defense, Tabuk, SAU; 2 Family Medicine, Prince Sultan Military Medical City, Riyadh, SAU; 3 Family Medicine, Ministry of Defense, Dhahran, SAU

**Keywords:** aviation safety, caffeine, fatigue countermeasures, military pilots, modafinil, operational performance, systematic review

## Abstract

Fatigue poses a significant risk to operational safety and performance in military aviation due to irregular schedules, night operations, and deployment environments. A range of countermeasures is employed, but their efficacy, specifically for military pilots, requires further exploration. This systematic review evaluated the current evidence on the effectiveness of behavioral, operational, and pharmacological fatigue countermeasures specifically for military pilots, to inform practice and policy. A systematic search of PubMed, Scopus, ScienceDirect, Research Gate, Cochrane Library, Google Scholar, and Web of Science was conducted for studies published between 2014 and 2025. Eligible studies included randomized controlled trials, non-randomized trials, and observational studies involving active-duty military pilots or aircrew, assessing any fatigue countermeasure against alertness, cognitive performance, or operational safety outcomes. Seven studies were included. Pharmacological interventions, particularly caffeine and modafinil, were the most effective short-term countermeasures, improving vigilance, reaction time, and performance during extended wakefulness. However, their efficacy was moderated by individual factors, such as innate fatigue vulnerability and sleep quality, and neither fully restored baseline performance under conditions of severe sleep deprivation (e.g., >27 hours awake). Evidence for behavioral countermeasures, such as habitual video gaming, was limited and showed no protective effect against cognitive fatigue. Technological approaches, like eye-tracking for real-time cognitive load monitoring, showed promise but require further validation in operational settings. This review shows that pharmacological strategies currently provide the most evidence-based approach for mitigating acute fatigue in military pilots. However, an over-reliance on stimulants is insufficient. Future fatigue management requires an integrated, personalized system combining real-time monitoring, validated countermeasures (including novel approaches and sleep-focused strategies), and a systemic cultural shift that prioritizes sleep as a critical component of operational readiness. Further high-fidelity research in real-world operational environments is essential.

## Introduction and background

Fatigue is a critical issue impacting military pilots, posing significant risks to operational safety (the capacity to maintain safe flight operations without error or incident) and mission effectiveness [1-3]. In aviation, fatigue impairs key aspects of situational awareness (the continuous perception and understanding of flight conditions and potential hazards) and cognitive performance (mental processes, such as attention, reaction time, memory, and decision-making, which are essential for safe flying) [1-3].

Military aviation demands high levels of alertness, situational awareness, cognitive function, and physical stamina, all of which are impaired by fatigue [1,2].

Unlike civilian aviation, military pilots operate under conditions that inherently increase fatigue risk, including irregular and extended duty hours, night operations, high-altitude missions, and deployment environments with limited rest opportunities. These factors compromise sleep quality and duration, leading to chronic fatigue accumulation. Empirical data show that over 60% of military pilots report moderate to severe fatigue during operations, and fatigue has been implicated in up to 20% of aviation-related incidents and human factor errors in military contexts [1-3].

Recent meta-analyses confirm that sustained sleep restriction and circadian disruption substantially impair attention, reaction time, working memory, and decision-making, which are core components of cognitive performance and situational awareness [4,5]. These effects are particularly pronounced in operational environments that demand prolonged wakefulness and rapid response under stress, such as military aviation, where fatigue has been consistently linked to degraded flight performance and safety outcomes [6,7]. Sleep opportunities in the field can be limited and of poor quality, necessitating effective fatigue countermeasures to sustain pilot fitness and flight safety [7,8]. This creates a critical need for both systemic and individualized strategies to manage fatigue in military aviation environments.

Fatigue countermeasures in military aviation encompass a range of behavioral, operational, and pharmacological interventions designed to mitigate the adverse effects of fatigue [2,9]. Key non-pharmacological approaches include regulated flight and duty time limitations, strategic scheduling, controlled napping (such as cockpit naps), and optimized rest facilities like bunk sleep. Additionally, active in-flight measures, such as activity breaks and adjustment of cockpit lighting, have been explored to enhance alertness [2]. Military forces also employ pharmacological aids like hypnotics to improve sleep and stimulants to maintain vigilance, although their use involves careful consideration of efficacy, safety, and operational constraints [2,10].

Despite advancements in understanding fatigue and developing countermeasures, implementing effective fatigue management in military aviation remains challenging due to the complexity of missions, diverse aircraft types, and operational demands [2,11]. Flight time limitations may be adjusted or overridden due to mission necessity, which underscores the importance of supplementary fatigue management strategies [2]. Scientific evaluation of these countermeasures is essential to ensure they reliably enhance performance and safety without unintended consequences.

This systematic review aims to examine the current evidence on the effectiveness of fatigue countermeasures specifically for military pilots. It will assess behavioral, operational, and pharmacological measures through a thorough analysis of available studies, identifying gaps and potential advancements in fatigue management in military aviation. The findings would help inform policy, operational practices, and future research directions to optimize the safety and performance of military aircrew operating under demanding conditions.

## Review

Methods

Study Design

This systematic review was conducted to synthesize and evaluate the current evidence on the effectiveness of behavioral, operational, and pharmacological fatigue countermeasures for military pilots, following the Preferred Reporting Items for Systematic Reviews and Meta-Analyses (PRISMA) guidelines [12].

The review was initiated by formulating a focused research question using the PICO (Population, Intervention, Comparison, Outcome) framework: What is the effectiveness of behavioral, operational, and pharmacological fatigue countermeasures in improving alertness, cognitive performance, and operational safety among military pilots?

Search Strategy

A systematic search strategy was executed to identify all relevant published literature. Electronic databases, including PubMed, Scopus, ScienceDirect, ResearchGate, Cochrane Library, Google Scholar, and Web of Science, were searched to identify peer-reviewed studies.

The search strategy employed a combination of controlled vocabulary (e.g., MeSH terms) and keywords related to three core concepts: (“military pilot” OR “aircrew”), (“fatigue” OR “sleep deprivation”), and (“countermeasure” OR “modafinil” OR “caffeine” OR “nap”). The reference lists of included studies and relevant review articles were also hand-searched to identify any additional eligible publications.

Search Period

The review included studies published between January 2014 and September 2025, and this 10-year timeframe ensured the inclusion of recent and updated evidence on recent interventions to mitigate fatigue. The last search was performed on 29 September 2025.

Study Selection

Eligibility criteria: The study selection followed predefined eligibility criteria based on the PICOS framework.

P - The population of interest was defined as active-duty military pilots or military aircrew members undergoing training. This included pilots from any branch of the armed forces (e.g., Air Force, Navy, Army Aviation) and any aircraft type (e.g., fighter, transport, rotary-wing). Studies focusing solely on civilian pilots, non-pilot military personnel, or general populations were excluded to maintain focus on the unique operational demands and physiological challenges faced in military aviation.

I - Eligible interventions included any pharmacological, behavioral, operational, or technological strategy explicitly implemented as a fatigue countermeasure. Pharmacological interventions of interest were stimulants (e.g., modafinil, caffeine, and amphetamines) and other alertness-promoting or sleep-enhancing compounds (e.g., hypnotics, galantamine). Behavioral and operational interventions included controlled napping (e.g., cockpit naps), structured sleep schedules, rest facilities, and cognitive training. Technological interventions involved real-time fatigue monitoring systems, such as eye-tracking devices. There were no restrictions on dosage, timing, or duration of the intervention.

C - The comparators considered were placebo controls, standard operational practice (i.e., no specific intervention), or baseline measurements within the same subjects (pre-intervention). This allowed for the assessment of the intervention's efficacy relative to a neutral control or the individual's typical performance state.

O - The primary outcomes were objective and subjective measures related to alertness, cognitive performance, and operational safety. Key metrics included psychomotor vigilance task (PVT), performance (reaction time, lapses), flight simulator performance (e.g., accuracy in maintaining altitude/heading), measures of subjective sleepiness (e.g., Stanford Sleepiness Scale), and in-flight performance metrics. Outcomes also included any reported side effects or operational limitations associated with the countermeasures.

S - Eligible study designs included randomized controlled trials (RCTs), non-randomized controlled trials, and comparative observational studies (cohort, case-control). Case reports, narrative reviews, editorials, conference abstracts, non-primary research, and non-comparative studies were excluded to ensure the synthesis was based on robust empirical evidence.

**Figure 1:**
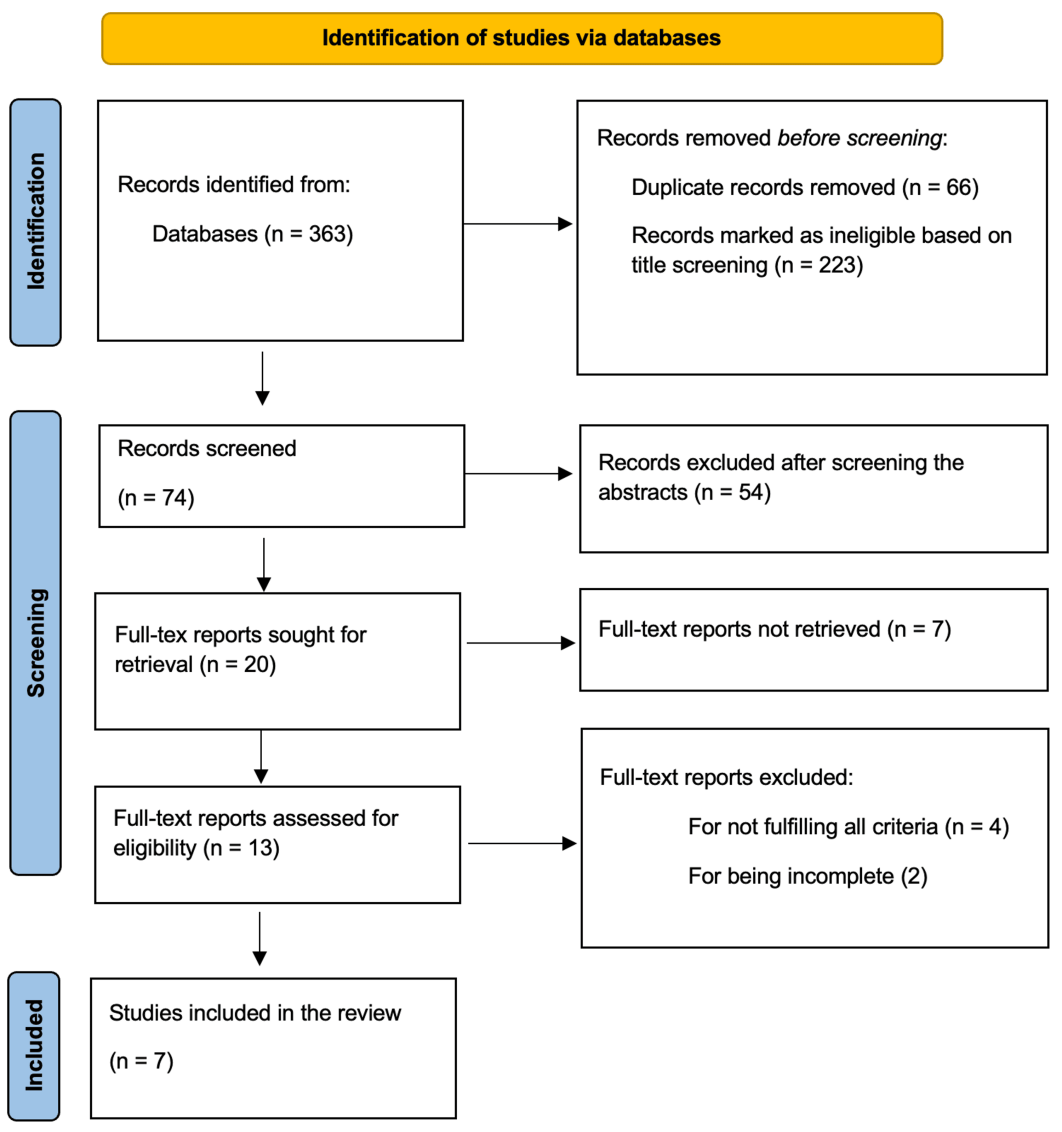
PRISMA flow chart showing the study selection process PRISMA: Preferred Reporting Items for Systematic Reviews and Meta-Analyses

Study screening: A two-stage screening process was then implemented. First, two independent reviewers screened titles and abstracts against pre-defined eligibility criteria. Full-text articles of potentially relevant studies were then retrieved and assessed in detail by the same reviewers. To be included, studies had to be original research articles published in English, involve a military pilot population, and empirically assess the effect of a fatigue countermeasure on alertness, cognitive performance, and operational safety. Disagreements between reviewers at any stage were resolved through discussion or by consulting a third reviewer. A PRISMA flow diagram was used to document the selection process (Figure 1).

Data Extraction and Quality Assessment

Data extracted from the included studies included: (1) study characteristics (authors, year, title, design); (2) participant demographics (sample size, age, military branch); (3) intervention details (type, dosage, timing); (4) comparator; (5) outcome measures and results; and (6) key conclusions. The risk of bias and quality of each included study were critically appraised using appropriate tools, such as the Cochrane Risk of Bias tool (RoB2; The Cochrane Collaboration, London, UK) [13] for randomized controlled trials and the Newcastle-Ottawa Scale [14] for observational studies. The Cochrane RoB2 assesses bias arising from the randomization process, deviations from intended interventions, missing outcome data, measurement of the outcome, and selection of the reported result. The NOS, on the other hand, assesses three domains: selection of the study groups (0-4 stars), comparability of groups based on design or analysis (0-2 stars), and outcome ascertainment (for cohort studies) or exposure measurement (for case-control) (0-3 stars).

Narrative Synthesis

Narrative synthesis was performed due to the anticipated heterogeneity in interventions, populations, and outcome measures, which precluded a meta-analysis. The findings were organized by countermeasure type (pharmacological, behavioral, technological) and summarized in evidence tables. The overall strength of the evidence for each countermeasure was graded based on the study design, sample size, methodological rigor, and consistency of findings, providing a clear overview of the current state of knowledge and its limitations.

Results

Table 1 shows that the seven included studies were characterized by diverse methodologies and specific military aviation populations. The research designs included three randomized controlled trials (RCTs), three observational studies, and one simulation-based mixed-method study, primarily focusing on distinct groups such as Royal Netherlands Air Force fighter pilots, military pilot students from Thailand, and members of the Indian Air Force. A notable consistency across the included research is the relatively small sample sizes, with participant numbers varying from 12 to 75, reflecting the common logistical challenges of conducting research within specialized military operational environments. 

**Table 1:** Characteristics of included studies FVUL – Fatigue-vulnerable, FINT – Fatigue-intermediate, FRES – Fatigue-resistant, stdev – Standard deviation

Study	Title	Study Design	Population Characteristics	Sample Size
Babu et al. [15], 2019	Estimating Pilots’ Cognitive Load From Ocular Parameters Through Simulation and In-Flight Studies	Simulation study (Controlled environment with a fixed-base variable-stability flight simulator); In-flight study (Observational design with structured test flights)	Indian Air Force pilots (Sqn Ldr to Gp Capt); mean age 35.4 ± 4.3 yrs; mean flying hours ≈ 1,766 ± 752	n = 14
Wingelaar-Jagt et al. [16], 2022	Subjective Effects of Modafinil in Military Fighter Pilots During Deployment	Field study, observational, non-controlled	Royal Netherlands Air Force fighter pilots	n = 75
Utamatanin et al. [17], 2022	The Effect of Caffeine and Sleep Quality on Military Pilot Students’ Flight Performance-Related Cognitive Function	Observational, within-subjects crossover, non-controlled, single-site	Military pilot students (Thailand); healthy males, mean age 25.1 years	n = 29
Wingelaar-Jagt et al. [18], 2023	Daily Caffeine Intake and the Effect of Caffeine on Pilots’ Performance After Extended Wakefulness	Randomized, double-blind, crossover, placebo-controlled trial	Aeromedically fit subjects engaged in military aviation	n = 30
Leelartapin et al. [19], 2023	Cognitive Fatigue in Habitual Video Gamers and Non-Gamers Among Military Pilots in Training	Controlled study, non-randomized, parallel design	Young male military pilots undergoing advanced flight training; age 24 ± 1 years (18 gamers, 16 non-gamers)	n = 34
Wingelaar-Jagt et al. [20], 2024	Comparison of Effects of Modafinil and Caffeine on Fatigue-Vulnerable and Fatigue-Resistant Aircrew After Limited Sleep Deprivation	Randomized, controlled, double-blind, placebo-controlled, parallel design	Royal Netherlands Air Force employees (66 % pilots); ages 25–59 yrs (median 30.9); 16 % female	n = 32( FVUL = 11; FINT = 10; FRES = 11)
Lewkowicz et al. [21], 2025	Flight Performance Following Administration of an Anti-Fatigue Stimulant During 27 Hours of Continuous Wakefulness	Randomized, placebo-controlled, crossover trial	Male military pilots; mean age 24 ± 2.5 years	n = 12

The evidence from the included studies demonstrates that fatigue countermeasures in military pilots vary in their effectiveness, depending on the type of intervention (pharmacological, behavioral, or technological), the context, and individual susceptibility to fatigue (Table 2).

**Table 2:** Effectiveness of fatigue countermeasures in military pilots G – Gravitational force (load factor in aviation/flight physiology), PVT – Psychomotor Vigilance Task, SSS – Stanford Sleepiness Scale

Study	Intervention effects	Summary of Main Findings
Wingelaar-Jagt et al. [16], 2022	Side effects: 1 out of 75 pilots experienced duty-relevant side effects. - Use in operational flights: 192 flights, with 67% used preventively and 33% due to fatigue. Positive effects: 95% of flights reported positive effects, with maximum effect 2-3 hours after administration. Impact on sleep quality: No negative effect on sleep quality. No correlation with caffeine or sleep medication.	Modafinil was used in 192 operational flights, mostly during night-time, with 67% used preventively and 33% due to fatigue. Positive effects of modafinil were reported in 95% of flights, with maximum effect 2-3 hours after administration. Modafinil did not negatively affect sleep quality or interact with caffeine or sleep medication, indicating its suitability as a countermeasure for fatigue in military aviation.
Lewkowicz et al. [21] 2025	Maintaining altitude: Galantamine: Improved performance under land and sea conditions (p<0.001) - Modafinil: No significant improvement, Placebo: Baseline for comparison - Speed: - Galantamine: Slower speeds compared to placebo - Modafinil: No significant effect – Placebo: Baseline for comparison - Maintaining stable heading: - Galantamine: Improved performance under sea conditions - Modafinil: No significant improvement Placebo: Baseline for comparison - Flight accuracy: - Galantamine: Did not restore to baseline levels - Modafinil: Did not restore to baseline levels - Placebo: Baseline for comparison	Galantamine showed significant improvements in maintaining altitude and stable heading, but resulted in slower speeds. Neither modafinil nor galantamine restored flight accuracy to baseline levels after 27 hours of wakefulness. The overall effects of both agents on sleep deprivation-induced fatigue and flight performance were minimal and comparable to the placebo.
Utamatanin et al. [17], 2022	Vigilance: Improved significantly after low-dose caffeine intake. Situational awareness: Improved significantly after low-dose caffeine intake. Reaction time: Improved significantly after low-dose caffeine intake. High sleep quality group: Improved vigilance and reaction time after caffeine intake. Low sleep quality group: Improved vigilance only after caffeine intake.	Low-dose caffeine intake significantly improved cognitive performance in vigilance, situational awareness, and reaction time. The cognitive-enhancing effect of caffeine was more pronounced in participants with high-quality sleep. Caffeine improved vigilance in both high and low sleep quality groups, but reaction time improvement was only seen in the high sleep quality group.
Wingelaar-Jagt et al. [20], 2024	PVT mean reaction time: - Placebo: Not mentioned - Modafinil: Significant improvement (p < 0.001) - Caffeine: Significant improvement (p < 0.001). PVT number of lapses: - Placebo: Not mentioned - Modafinil: Significant improvement (p = 0.036) - Caffeine: Non-significant improvement (p = 0.130). SSS: - Placebo: Not mentioned - Modafinil: Significant improvement (p < 0.001) - Caffeine: Significant improvement (p < 0.001) - VigTrack mean reaction time: - Placebo: Not mentioned - Modafinil: Significant improvement (p = 0.036) - Caffeine: Significant improvement (p = 0.009). VigTrack mean percentage omissions: - Placebo: Not mentioned - Modafinil: Not significant - Caffeine: Not significant - VigTrack mean tracking error: - Placebo: Not mentioned - Modafinil: Not significant - Caffeine: Not significant	The study found significant effects of modafinil and caffeine on improving performance in fatigue-vulnerable individuals. Fatigue-intermediate and fatigue-resistant individuals performed better than fatigue-vulnerable individuals in several parameters. The effectiveness of stimulants varies depending on an individual's fatigue tolerance, with fatigue-vulnerable individuals benefiting more from stimulant administration.
Babu et al. [15], 2019	Rate of fixation: - Simulation study: Significantly different in various flying conditions (p<0.05) - In-flight study: Higher during take-off, landing, and maneuvering stages (p<0.001) - Correlated with rate of descent during air to ground dives (r>0.7, p<0.05) - Correlated with normal load factor (G) during constant G level turns - Number of saccades: - Simulation study: Significantly different in various flying conditions (p<0.05) - Pupil dilation: - Simulation study: Significantly different in various flying conditions (p<0.05) - In-flight study: Not significantly different due to variable lighting conditions	Commercial eye gaze tracking glasses can be used to measure ocular parameters in combat aircraft up to +6G. The rate of fixation is measured using the algorithm discussed increases for tasks demanding a higher cognitive workload from pilots. All pupil dilation-based metrics should be tested in variable lighting and vibrating conditions of an actual aircraft before using them for cognitive load estimation.
Wingelaar-Jagt et al. [18], 2023	Performance decrease during the night: - Caffeine condition: Decreased significantly less than placebo - Placebo condition: Not specified – Habitual intake and daytime caffeine consumption: No effect on performance - Blood concentrations of caffeine and performance: No statistically significant correlation	A single dose of 300 mg of caffeine has beneficial effects on performance during the night in a realistic scenario for military aviation. Neither habitual intake nor daytime caffeine consumption affects the effects of caffeine at night. No statistically significant correlation was identified between blood concentrations of caffeine and performance.
Leelartapin et al. [19] 2023	Reaction time (PVT): Increased significantly (p < 0.05) in both groups. - Spatial ability reaction time (mental rotation task): Decreased significantly (p < 0.05) in both groups. Time estimation accuracy (time-wall task): No significant changes were observed in either group. - Spatial ability accuracy (mental rotation accuracy): No significant changes in both groups. Time-averaged mean blood flow velocity: Remained unchanged in both groups. - Cognitive function and brain perfusion: No apparent differences between habitual video gamers and non-gamers.	Both habitual video gamers and non-gamers showed increased reaction times in the psychomotor vigilance task and decreased reaction times in the mental rotation task after cognitive fatigue induction. No significant changes were observed in time estimation accuracy or spatial ability accuracy. There were no differences in brain perfusion between the groups during the tasks.

Pharmacological Countermeasures

Pharmacological interventions were the most widely studied fatigue countermeasures among military pilots, and they were reported by five included articles. Modafinil demonstrated operational effectiveness, with 95% of flights reporting positive effects during deployment and only one case of duty-relevant side effects. Importantly, modafinil did not negatively impact sleep quality or interact with caffeine or sleep medications, making it a suitable countermeasure in operational contexts [16]. However, its efficacy under severe sleep deprivation was more limited. In a placebo-controlled crossover trial, neither modafinil nor galantamine restored flight performance to baseline after 27 hours of continuous wakefulness. While galantamine improved altitude and heading maintenance, it resulted in slower speeds, and both agents failed to restore flight accuracy [21].

Caffeine showed more consistent benefits. Low-dose caffeine significantly improved vigilance, situational awareness, and reaction time in pilot students, with greater effects observed in those with high-quality sleep [17]. A single 300 mg caffeine dose also mitigated nighttime performance declines in a realistic operational scenario, irrespective of habitual intake or daytime caffeine use [18]. A direct comparison of caffeine and modafinil revealed that both enhanced psychomotor vigilance and reduced sleepiness, but their effects varied by individual fatigue vulnerability. Fatigue-vulnerable pilots derived the most significant performance improvements, suggesting inter-individual differences in stimulant efficacy [20].

Overall, stimulant countermeasures are effective for maintaining vigilance and cognitive performance in the short term, but they cannot fully restore baseline function after extended wakefulness. Their benefits also appear to be moderated by sleep quality and individual fatigue resistance.

Behavioral and Cognitive Countermeasures

Evidence for behavioral countermeasures remains limited since it was reported on by one included study. A controlled study investigated the potential protective role of habitual video gaming on cognitive fatigue and brain perfusion in trainee pilots. Both gamers and non-gamers showed similar declines in psychomotor vigilance and spatial ability following cognitive fatigue induction, with no significant differences in brain perfusion between groups [19]. This suggests that video gaming experience does not attenuate the impact of fatigue on cognitive performance in military pilot trainees.

Technological Innovations and Monitoring Approaches

One study in our review reported on the technological innovations and monitoring approaches. Emerging technological approaches aim to monitor fatigue and cognitive load in real time. Eye-tracking studies demonstrated that ocular parameters, such as fixation rates and saccadic movements, correlated with task demand and flight maneuvers in both simulation and in-flight conditions. While pupil dilation metrics were less reliable under variable lighting, fixation-based measures provided meaningful insights into workload. These findings highlight the potential of eye-tracking technologies to serve as early warning tools for cognitive overload in military aviation, enabling timely activation of countermeasures [15].

Summary and Strength of Evidence

The evidence map highlights that pharmacological countermeasures, particularly caffeine and modafinil, provide the most consistent benefits for mitigating fatigue in military pilots, improving vigilance, reaction time, and performance during extended wakefulness. However, neither fully restores baseline functioning under severe sleep deprivation. Their effects appear stronger among pilots with high-quality sleep and those more vulnerable to fatigue. By contrast, behavioral approaches, such as habitual video gaming, show no protective effect on cognitive fatigue or brain perfusion, while technological innovations like eye-tracking offer promising real-time monitoring tools for detecting cognitive overload but remain exploratory. Overall, stimulant-based interventions carry the strongest evidence base, whereas behavioral and technological strategies are less established, underscoring the need for further operationally embedded studies to validate long-term effectiveness and safety (Table 3).

**Table 3:** Evidence on fatigue countermeasures in military pilots RCT – Randomized controlled trial

Countermeasure	Population / Context	Main Outcomes	Strength of Evidence
Modafinil [16]	Royal Netherlands Air Force pilots, operational flights	95% flights positive effects; no adverse impact on sleep; suitable operational use	Moderate (field study, large sample, but non-controlled)
Modafinil and Galantamine [21]	Military pilots, 27h continuous wakefulness	Minimal restoration of flight performance; galantamine improved altitude/heading but slowed speed	Moderate (small RCT, strong design but very small sample)
Caffeine [17]	Military pilot students in Thailand	Improved vigilance, situational awareness, and reaction time; stronger in high sleep quality pilots	Moderate (observational, small sample, crossover)
Caffeine [18]	Military aviation scenario, night operations	Reduced performance decline at night; benefits independent of habitual intake	High (randomized, double-blind, placebo-controlled crossover)
Caffeine vs Modafinil [20]	Royal Netherlands Air Force, fatigue-vulnerable vs resistant	Both stimulants improved reaction time and vigilance; fatigue-vulnerable pilots benefited most	High (randomized, placebo-controlled, parallel groups)
Video Gaming [19]	Pilot trainees, advanced training	No differences between gamers and non-gamers; cognitive fatigue unaffected	Low (non-randomized, small sample)
Eye-tracking [15]	Indian Air Force pilots, simulator & in-flight	Fixation/saccade metrics correlated with workload; potential for real-time monitoring	Low-Moderate (simulation + in-flight, small sample, exploratory)

Quality Assessment of Included Studies

The most robust and reliable findings come from high-quality RCTs with a low risk of bias, which consistently demonstrate the short-term efficacy of pharmacological countermeasures like caffeine and modafinil in improving alertness and performance. In contrast, the evidence for behavioral and technological interventions is considerably weaker, derived from studies with a moderate to high risk of bias due to non-randomized designs, small samples, and a lack of control for confounding variables (Table 4). Consequently, while pharmacological strategies are well-supported, conclusions about the effectiveness of other countermeasures remain preliminary, underscoring a critical need for more methodologically rigorous, operationally relevant research in these areas.

**Table 4:** Quality/risk of bias of included studies RoB 2 – Cochrane Risk of Bias 2 tool, NOS – Newcastle–Ottawa Scale

Study	Study Design	Assessment Tool	Key Quality Findings	Overall Quality / Risk of Bias
Lewkowicz et al. [21], 2025	Randomized Controlled Trial (RCT)	RoB 2	Low risk in randomization, blinding, and reporting; *some concerns* in the selection of reported results due to a very small sample (n = 12) and a possible performance bias in the crossover design.	Some Concerns / Moderate (Strong design but very small sample size)
Wingelaar-Jagt et al. [20], 2024	Randomized Controlled Trial (RCT)	RoB 2	Low risk in randomization, blinding (double-blind, placebo-controlled), and reporting; rigorous parallel-group design.	High Quality / Low Risk
Wingelaar-Jagt et al. [18], 2023	Randomized Controlled Trial (RCT)	RoB 2	Low risk in randomization, blinding (double-blind, placebo-controlled, crossover), and reporting; robust methodology.	High Quality / Low Risk
Wingelaar-Jagt et al. [16], 2022	Observational Field Study	NOS	Representative operational pilot sample, but selection not described; no control group; self-reported outcomes introduce reporting bias.	Moderate Risk (Non-controlled design limits causality despite large sample)
Utamatanin et al. [17], 2022	Observational (Within-Subjects)	NOS	Defined group of pilot students; within-subjects design controls for individual differences but lacks randomization and blinding.	Moderate Risk (Crossover design strong, but limited control and blinding)
Leelartapin et al. [19], 2023	Non-Randomized Controlled Trial	NOS	Groups formed by habit (gaming vs. non-gaming), introducing selection bias; possible confounders; objective measures, but not blinded.	High Risk (Major limitations due to non-randomization and confounding)
Babu et al. [15], 2019	Mixed-Methods Simulation + In-Flight (Observational)	NOS	Small specific sample; no control group; objective metrics used, but real-world flight conditions introduce uncontrolled confounders (lighting, vibration).	High Risk (Exploratory, small sample, limited control)

Discussion

The findings of this systematic review underscore the complex and multifaceted nature of fatigue management in military aviation. Pharmacological interventions, particularly caffeine and modafinil, emerged as the most consistently effective short-term countermeasures for sustaining alertness and cognitive performance during extended wakefulness. However, their efficacy is not absolute; under circumstances of extreme sleep loss, such as following 27 hours of nonstop alertness, neither compound completely restores baseline performance [21]. This aligns with broader literature indicating that stimulants are effective for mitigating acute fatigue but cannot replace the fundamental need for sleep [22,23]. The moderating effects of sleep quality and individual susceptibility further highlight that pharmacological strategies are not one-size-fits-all solutions [17,20]. In contrast to the relative robustness of pharmacological evidence, behavioral countermeasures yielded less promising results. The study by Leelartapin et al. found that habitual video gaming offered no protective effect against cognitive fatigue in trainee pilots, challenging the notion that certain leisure activities could build cognitive resilience [19]. This finding is significant as it suggests that not all cognitively engaging tasks transfer to fatigue resistance in high-stakes operational environments. Other behavioral strategies, such as strategically timed naps or rest breaks, have shown more promise in non-aviation populations. For instance, Watling et al. demonstrated that nap breaks were more effective than active rest breaks for reducing physiological sleepiness in drivers [24]. Furthermore, cognitive behavioral therapy for insomnia (CBT-I) has proven highly effective in improving sleep and reducing fatigue in military personnel and other shift workers, addressing the root cause of fatigue rather than its symptoms [25,26]. The limited exploration of such sleep-focused behavioral interventions within the specific context of in-flight military operations represents a considerable gap in the current research landscape.

Technological innovations for real-time fatigue monitoring, such as eye-tracking, present a forward-looking avenue for fatigue management. Babu et al. demonstrated that ocular parameters can provide reliable metrics of cognitive load during flight, offering the potential for just-in-time interventions [15]. This aligns with developments in other domains, and Jackson et al. found that automated ocular metrics were effective predictors of driver drowsiness [27]. The integration of such systems into cockpit avionics could enable a paradigm shift from reactive to proactive fatigue management, allowing for interventions to be deployed before performance degradation reaches critical levels [28,29].

When contextualized within the broader literature presented in Table 4, several critical insights emerge. First, the effectiveness of any countermeasure is heavily dependent on the operational context. For example, Banks et al. emphasize the need for a “countermeasure toolbox” for 24/7 warfighters, integrating behavioral, operational, and pharmacological strategies tailored to maritime environments [30]. This holistic approach is arguably even more critical in military aviation, where mission demands are unpredictable and often override standardized duty-time limitations. Second, novel interventions like transcranial direct current stimulation (tDCS) show considerable promise. Previous studies found tDCS to be more effective than caffeine in sustaining vigilance and cognitive performance over extended periods [28,31], suggesting a potential non-pharmacological frontier for future research in aviation. However, its practical application in the cockpit remains to be explored. An important finding that goes beyond the type of countermeasure is the role of individual differences. Factors such as innate fatigue vulnerability [20], circadian chronotype [32], and baseline sleep quality [17] significantly moderate the efficacy of interventions. This underscores the necessity of moving beyond population-wide recommendations toward personalized fatigue risk management systems. The development of predictive tools like the 2B-Alert Web model [29], which forecasts neurobehavioral performance based on sleep history and caffeine consumption, exemplifies the kind of individualized approach required for future operational effectiveness.

Finally, the review highlights a significant disconnect between controlled studies and operational reality. While laboratory-based RCTs provide essential evidence on efficacy, research shows that military personnel consistently experience chronic sleep restriction and circadian disruption [33,34]. Therefore, the most effective countermeasure may ultimately be systemic and cultural (the one that prioritizes sleep as a critical component of operational readiness through leadership support, education, and evidence-based scheduling policies) [35,36].

This review has some limitations to consider. The most significant is the high degree of heterogeneity in the included studies, which varied in their interventions, populations, outcome measures, and, crucially, methodological quality, precluding a meta-analysis and necessitating a narrative synthesis. The evidence base was also limited by the small sample sizes common in military aviation research and a reliance on simulator-based or subjective outcomes, which may not fully translate to real-world operational performance and safety. Furthermore, the search was restricted to studies published in English, potentially introducing language bias, and the focus on military pilots, while specific, limits the generalizability of the findings to other military aircrew or civilian aviation contexts. Another key limitation of the present synthesis is the diverse statistical reporting across primary studies, as there were no standardized effect sizes or confidence intervals, restricting the ability to collectively quantify the magnitude of observed improvements. Future research should ensure standardized reporting to facilitate meta-analytic comparisons.

## Conclusions

This systematic review establishes that pharmacological interventions, particularly caffeine and modafinil, currently form the most evidence-based and effective strategy for mitigating acute fatigue and sustaining cognitive performance in military pilots during short-term sleep deprivation. However, their efficacy is moderated by individual factors like innate fatigue vulnerability and sleep quality. In contrast, evidence for behavioral and technological countermeasures remains nascent and less compelling. Therefore, the future of fatigue management in military aviation does not lie in a single solution but in an integrated, personalized system. This requires a synergistic combination of reliable real-time monitoring technology, validated countermeasures, including novel approaches like tDCS and structured sleep protocols, and a systemic cultural shift that prioritizes sleep as a critical component of operational readiness to ensure the safety and cognitive dominance of military pilots. Future research should prioritize high-fidelity, operational studies conducted in real-world military settings to validate countermeasures beyond simulated environments. Investigations must adopt personalized approaches, identifying biomarkers or traits that predict individual efficacy to move beyond one-size-fits-all solutions. Finally, studies should focus on integrated systems that combine technological monitoring with targeted interventions to enable proactive, real-time fatigue management. Moreover, future research should prioritize large-scale, multicenter randomized trials conducted under real operational conditions, integrating both pharmacological and behavioral interventions. Standardization of fatigue and performance measures, coupled with exploration of long-term effects and individualized fatigue profiles, will be critical to developing robust, personalized fatigue management systems in military aviation.
